# Emotional blunting in patients with depression. Part IV: differences between patient and physician perceptions

**DOI:** 10.1186/s12991-022-00391-5

**Published:** 2022-06-22

**Authors:** Michael Cronquist Christensen, Hongye Ren, Andrea Fagiolini

**Affiliations:** 1 H. Lundbeck A/S, Medical Affairs, Ottiliavej 9, 2500 Valby, Denmark; 2 Division of Psychiatry, Department of Molecular and Developmental Medicine, University of Siena School of Medicine, Siena, Italy

**Keywords:** Depression, Emotional blunting, Functioning, Healthcare provider perspectives, Oxford Depression Questionnaire, Patient perspectives, Treatment phase

## Abstract

**Background:**

Emotional blunting is common in patients with depression. An online survey was undertaken to assess the experience of emotional blunting, and its impact on functioning and quality of life, in the acute and remission phases of depression from the perspective of patients and healthcare providers (HCPs). This paper presents data on the level of concordance between patient and HCP perspectives.

**Methods:**

This was a cross-sectional, observational study. Patient respondents were adults with a diagnosis of depression, who were currently using a prescribed antidepressant, and who reported emotional blunting during the past 6 weeks. HCPs completed the survey for the last two eligible patients they had seen, one in each phase of depression. Assessments included the Oxford Depression Questionnaire (ODQ) ‘antidepressant as cause’ domain and the Functioning Assessment Short Test (FAST).

**Results:**

Mean ODQ ‘antidepressant as cause’ domain scores were significantly higher in the patient-reported cohort (*n* = 752) than in the HCP-assessed cohort (*n* = 766) in both the acute (18.0 vs 12.5, respectively; *p* < 0.01) and remission phases (17.6 vs 12.6; *p* < 0.01). Overall, 45% of patients believed that their antidepressant medication was negatively affecting their emotions and 39% were considering stopping or had stopped their antidepressant because of perceived emotion-related side effects. In the HCP-assessed cohort, the antidepressant was considered responsible for emotional blunting in 30% of patients and only 18% of patients were believed to be considering stopping their medication due to emotional blunting. Patients reported a greater impact of emotional blunting on activities of daily living than HCPs. Mean FAST score was significantly higher in each phase of depression in the patient-reported cohort than in the HCP-assessed cohort (acute phase, 47.0 vs 39.1; remission phase, 33.5 vs 19.4; both *p* < 0.01).

**Conclusions:**

Compared with previous studies, our results suggest that HCPs may underestimate the prevalence of emotional blunting in patients with depression. HCPs also appear to underestimate the severity and impact of emotional blunting on patient functioning and treatment adherence compared with patients’ own perspectives. Differences between patient and HCP perspectives were most pronounced during the acute phase of the disease.

**Supplementary Information:**

The online version contains supplementary material available at 10.1186/s12991-022-00391-5.

## Introduction

Emotional blunting is a common symptom in patients with depression, being reported in about 60% of patients receiving selective serotonin reuptake inhibitors (SSRIs) or serotonin-noradrenaline reuptake inhibitors (SNRIs) [1–7]. Patients experiencing emotional blunting report feeling a restricted range of emotions and an inability to experience expected emotional responses [5]. Anhedonia (the inability to anticipate and experience pleasure) is one aspect of emotional blunting in patients with depression and is one of the core diagnostic criteria for a major depressive episode [8]. Although there is overlap between emotional blunting and anhedonia, these symptoms are not identical.

Emotional blunting is clinically important and burdensome for patients; for example, patients may report changes in their personality, reduced inspiration or motivation, general indifference to events and people that should matter to them, or a negative impact on decision-making, judgment, and relationships [1, 6]. The impact of emotional blunting on decision-making can also affect treatment adherence and therefore potentially increase the risk of relapse [9]. In one study of 316 patients with major depressive disorder, 35% discontinued treatment due to emotional blunting [10].

The importance of patient-centered care in managing mental health disorders such as depression is well-recognized [11–16]. However, studies have shown that differences exist between the perspectives of patients with depression and their healthcare providers (HCPs) regarding the importance and impact of symptoms experienced and treatment goals [17–21]. Data on HCP perspectives on the prevalence of emotional blunting and its impact on activities of daily living in patients with depression are lacking. A large online survey was undertaken to assess the experience of emotional blunting in the acute and remission phases of depression from the perspective of both patients and HCPs. Previous papers have presented data concerning the clinical characteristics of emotional blunting in depression [22], the impact of emotional blunting on overall functioning and quality of life in patients with depression [23], and its relationship with psychological trauma [24]. This paper—the last in the series—presents data on the level of concordance between patient and HCP perspectives regarding the experience and impact of emotional blunting in depression.

## Methods

### Study design and participants

This was a quantitative, cross-sectional, observational study conducted between April 15 and May 18, 2021, in Brazil, Canada, and Spain by BPR Pharma (London, UK). Data were collected through a self-completed online survey. Study design has been described in detail in the first paper in this series [22]. In brief, respondents were recruited through existing online panels of consumers and HCPs. Patients and HCPs were unmatched (i.e., patients who participated in this survey were not the same patients as those described by the HCPs).

Patient respondents were aged 18–70 years, had been diagnosed with depression by a physician, were currently taking a prescribed antidepressant, and reported emotional blunting in the last 6 weeks. Emotional blunting was described as follows: *‘Emotional effects of depression and treatment vary, but may include, for example, feeling emotionally “numbed” or “blunted” in some way; lacking positive emotions or negative emotions; feeling detached from the world around you; or “just not caring” about things that you used to care about.’* Quotas were imposed for patient age (≥ 50 years, 50%) and sex (female, 60%). Patients were required to be in the acute or remission phase of depression. The acute phase of depression was defined as: *‘A time when your symptoms are at their worst or most severe and for which you use antidepressant treatment.’ *Remission was defined as: *‘A time when your symptoms have improved significantly and you are already feeling better, but you may or may not still experience some minor symptoms. You are still taking antidepressant medication.’*

HCP respondents were psychiatrists or primary care physicians spending at least 75% of their time in direct patient care and seeing a minimum number of depressed patients a month [psychiatrists, 40; primary care physicians, 15 (10 in Brazil)], who were personally responsible for prescribing antidepressant medication to at least 75% of their patients with depression. For psychiatrists, quotas were imposed on hospital and office settings (each 50%). Patients assessed by HCPs were required to be aged 18–75 years, diagnosed with depression, and receiving antidepressant medication. HCPs completed the survey for two eligible patients: one in the acute phase of depression and one in remission from depression. The acute phase was defined as:* ‘A patient experiencing acute symptoms of depression that require antidepressant treatment.’* Remission was defined as: *‘The patient feels better and experiences a significant reduction in symptoms compared to other phases. Some residual symptoms may persist, but are significantly fewer in number and severity compared to other phases. The patient is still on antidepressant medication.’*

Informed consent was obtained from all respondents prior to screening. The study was approved by an institutional review board (Veritas IRB, Montreal, QC, Canada) and was conducted in accordance with the European Pharmaceutical Market Research Association (EphMRA) code of conduct, General Data Protection Regulation (GDPR), and all relevant local market laws.

### Assessments

In both surveys, respondents were asked which of a list of symptoms they (or their patient) had experienced during their phase of depression as defined in this survey. For HCPs, emotional blunting was included in this list, defined as: *‘Emotionally numb or blunted (lacking positive emotions or negative emotions; feeling detached from the world around; or “just not caring” about things that they used to care about).’* Symptom severity was rated on a scale of 1 (not at all severe) to 7 (extremely severe).

Both the patient and HCP surveys incorporated the Oxford Depression Questionnaire (ODQ) ‘antidepressant as cause’ domain and the Functioning Assessment Short Test (FAST). The ODQ is a validated instrument for assessing emotional blunting in patients with depression over the previous 7 days [25, 26]. Only the ‘antidepressant as cause’ domain was completed by both patients and HCPs. This domain is only relevant to patients currently receiving antidepressants for their depression, and explores their perception of a potential link between their current antidepressant and their experience of emotional blunting, and whether this has affected adherence to treatment. Respondents are asked the extent to which they agree or disagree with each statement; responses are indicated on a 5-point scale ranging from 1 (disagree) to 5 (agree). The maximum possible total score for the ‘antidepressant as cause’ domain is 30 points.

The FAST questionnaire comprises 24 questions that assess functioning across six domains: autonomy, occupational functioning, cognitive functioning, financial issues, interpersonal relationships, and leisure time [27]. For this survey, the period of recall was ‘during this acute or remission phase of depression.’ Respondents were asked to indicate the degree of difficulty experienced with each item (‘no difficulty’, ‘mild difficulty’, ‘moderate difficulty’, ‘severe difficulty’, or ‘don’t know’). The FAST total score ranges from 0 to 72, with higher scores indicating greater functional impairment.

Respondents also rated the impact of emotional blunting and anhedonia on different aspects of daily living (namely, their ability to function at work, in their home/family life, and in their social life) and on their overall quality of life. Impact of mood symptoms, cognitive symptoms, and fatigue/lack of energy on these aspects was also assessed. Impact was rated on a 7-point scale, with a score of 6 or 7 demonstrating significant impact.

### Statistical analysis

The analysis population comprised all respondents who met the study inclusion criteria and completed the online survey. Data were analyzed separately for patients and HCPs. In both cohorts, any respondent who responded ‘don’t know’ to more than one item for any FAST domain was excluded from the analysis of that domain. For respondents who responded ‘don’t know’ to just one item in any FAST domain, the mean score for the other items answered in that domain was used to impute the missing value (in line with FAST scale guidance). Respondents with a missing score for any domain were removed from the calculation of FAST total score.

Data are presented descriptively (means and standard deviations [SDs] for continuous variables, and frequencies and percentages for categorical variables). Comparisons were performed for continuous measures using *t* tests and for proportions using *Z* tests; significance was set at *p* < 0.05. Data were analyzed by The Stats People (Sevenoaks, UK) using Merlin tabulation software and Microsoft Excel.

## Results

### Patient demographics

A total of 752 patients and 383 HCPs (including 226 psychiatrists and 157 primary care physicians) completed the survey. HCPs provided data for a total of 766 patients. Baseline characteristics for the two patient cohorts are shown in Table 1. Sociodemographic characteristics were similar in the two patient cohorts. However, the mean duration of depression was longer in the patient-reported cohort than in the HCP-assessed cohort (62.4 vs 46.5 months). The proportion of patients who had experienced depression for more than 5 years was also higher in the patient-reported cohort (50% vs 19% in the HCP-assessed cohort). The proportion of patients reporting past or present drug or alcohol abuse in the patient-reported cohort was approximately double that reported in the HCP-assessed cohort, both overall and for each phase of depression. In both cohorts, most patients were currently receiving treatment with an SSRI or SNRI.

**Table 1:** Patient demographics and baseline characteristics

	Patient-reported cohort (*N* = 752)	HCP-assessed cohort (*N* = 766)
Country, *n* (%)		
Brazil	251 (33)	252 (33)
Canada	251 (33)	260 (34)
Spain	250 (33)	254 (33)
Sex, *n* (%)		
Female	466 (62)^a^	484 (63)
Age group, *n* (%)		
18–34 years	166 (22)^a^	189 (25)
35–54 years	382 (51)^a^	385 (50)
55–70 years	204 (27)^a^	192 (25)
Time since diagnosis of depression (months)		
Mean (standard deviation)	62.4 (49.5)	46.5 (72.7)
Depression phase, *n* (%)		
Acute	300 (40)	383 (50)^b^
Remission	452 (60)	383 (50)^b^
High school education or above, *n* (%)	596 (79)	631 (82)
Work status, *n* (%)		
Full-time	348 (46)	330 (43)
Part-time	93 (12)	88 (11)
Homemaker/stay at home parent	45 (6)	58 (8)
Student	22 (3)	39 (5)
Retired	89 (12)	68 (9)
Sick leave	43 (6)	113 (15)
Unemployed	112 (15)	70 (9)
In a relationship, *n* (%)	471 (63)	490 (64)
Ever addicted to drugs or alcohol, *n* (%)		
Overall	156 (21)	68 (9)
Acute	71 (24)	38 (10)
Remission	85 (19)	30 (8)

^a^For patient respondents, quotas were imposed for age (≥ 50 years, 50%) and sex (female, 60%)

^b^Each HCP completed the survey for two eligible patients: one in the acute phase of depression and one in remission from depression

HCP, healthcare provider

### Symptoms and severity

**Fig. 1:**
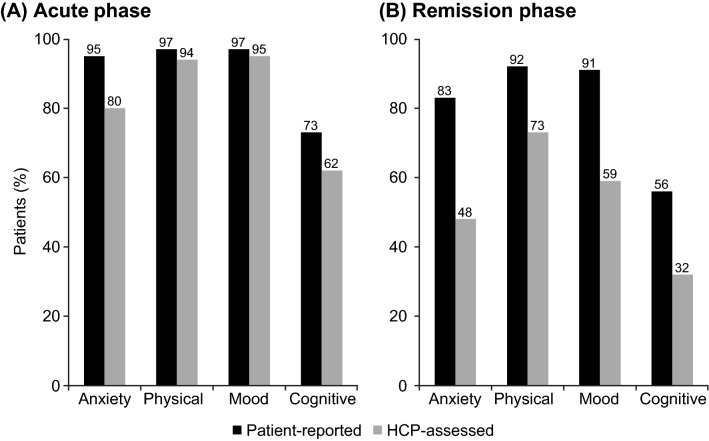
Symptoms reported by domain and phase of depression in the two patient cohorts. *p* < 0.01 for all differences between the acute and remission phases within each cohort. HCP, healthcare provider

In the acute phase of depression, the prevalence of anxiety and cognitive symptoms was higher in the patient-reported cohort than in the HCP-assessed cohort (95% vs 80%, respectively, for anxiety symptoms and 73% vs 62%, respectively, for cognitive symptoms). The prevalence of mood and physical symptoms in the acute phase of depression was broadly similar in the two cohorts (Fig. 1). In the remission phase, the prevalence of all symptom types was markedly higher in the patient-reported cohort than in the HCP-assessed cohort (Fig. 1).

**Fig. 2:**
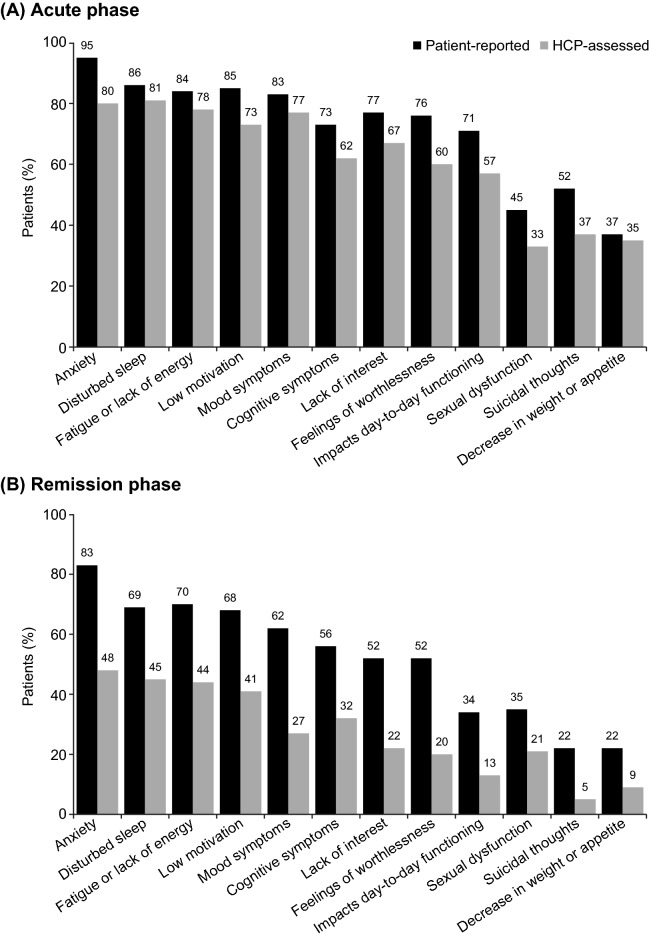
Individual symptoms reported in the two patient cohorts according to phase of depression. *p* < 0.01 for all differences between the acute and remission phases within each cohort. HCP, healthcare provider

In the acute phase of depression, the prevalence of all individual symptoms was also higher in the patient-reported cohort than in the HCP-assessed cohort, and these differences were even more marked in the remission phase of the disease (Fig. 2). Irrespective of the phase of depression, anxiety was the most frequently experienced symptom (reported by 95% of patients in the acute phase of depression and 83% of those in remission; corresponding rates in the HCP-assessed cohort were 80% and 48%). Other frequently reported symptoms in both cohorts were mood symptoms, disturbed sleep, fatigue/lack of energy, and low motivation in both phases of depression. Lack of interest (i.e., anhedonia) was reported by 77% of patients in the acute phase and 52% of those in remission; corresponding rates in the HCP-assessed cohort were 67% and 22%.

**Fig. 3:**
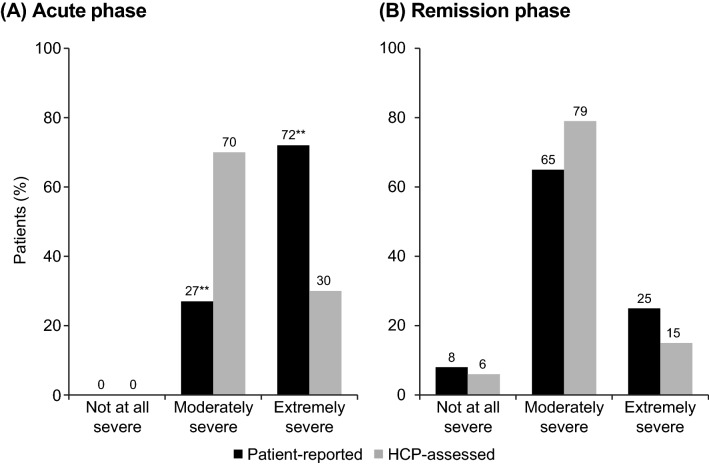
Severity of emotional blunting in the two patient cohorts according to phase of depression. **p < 0.01 for patient-reported vs HCP-assessed cohort within phase of depression. Patient-reported cohort: acute phase, n = 300; remission phase, n = 452. HCP-assessed cohort: acute phase, n = 122; remission phase; n = 52. HCP, healthcare provider

Per protocol, all patients in the patient-reported cohort had experienced emotional blunting within the past 6 weeks. In the HCP-assessed cohort, 23% of patients were reported to have experienced emotional blunting (32% of patients in the acute phase of depression and 14% of those in remission). Patients were significantly more likely than HCPs to report extremely severe emotional blunting (a score of 6 or 7 for emotional blunting was reported for 44% vs 26% of patients in the two cohorts, respectively; *p* < 0.01). Differences in the prevalence of extremely severe emotional blunting between the patient-reported and HCP-assessed cohorts were most apparent during the acute phase of depression (72% vs 30% in the two cohorts, respectively; *p* < 0.01) (Fig. 3).

**Fig. 4:**
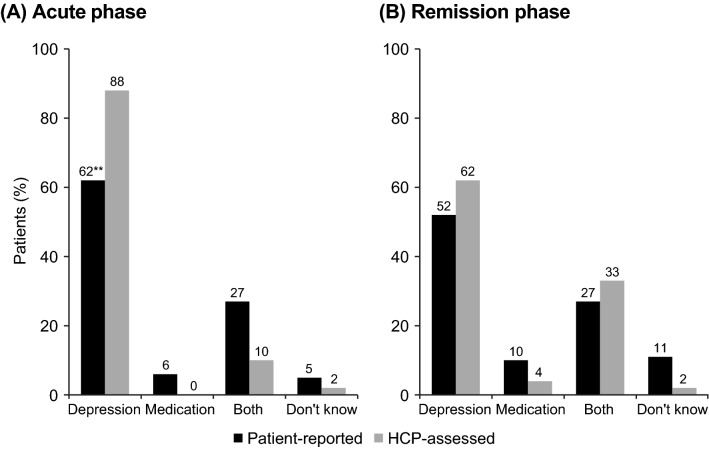
Perceived cause of emotional blunting in the two patient cohorts according to phase of depression. **p < 0.01 for patient-reported vs HCP-assessed cohort within phase of depression. Patient-reported cohort: acute, *n* = 300; remission, *n* = 452. HCP-assessed cohort: acute, *n* = 122; remission; *n* = 52. HCP, healthcare provider

Irrespective of the phase of depression, most respondents in both cohorts considered depression to be the main cause of emotional blunting (Fig. 4). In the acute phase of depression, 62% of patients and 88% of HCPs indicated that they considered depression to be the main cause of their/the patient’s emotional blunting (difference, *p* < 0.01). In the remission phase, depression was perceived to be the main cause of emotional blunting in 52% and 62% of patients in the two cohorts, respectively.

### ODQ and FAST questionnaire findings

Mean (SD) ODQ ‘antidepressant as cause’ domain scores were significantly higher in the patient-reported cohort than in the HCP-assessed cohort in both the acute phase (18.0 [6.4] vs 12.5 [5.3], respectively; *p* < 0.01) and the remission phase (17.6 [5.9] vs 12.6 [5.9], respectively; *p* < 0.01). In the patient-reported cohort, 45% of patients believed that their antidepressant medication was negatively affecting their emotions, while the antidepressant was considered responsible for emotional blunting in 30% of patients in the HCP-assessed cohort (Table 2). In all, 39% of patients indicated that they were considering stopping or had stopped their antidepressant due to its perceived emotion-related side effects, while HCPs believed only 18% of patients were considering stopping their medication due to potential emotion-related side effects.

**Table 2:** Patient and healthcare provider net agreement with the items of the ‘antidepressant as cause’ domain of the Oxford Depression Questionnaire

	Patient agreement^a^ (%)	HCP agreement^a^ (%)
The antidepressant is preventing me/the patient from feeling my/their emotions in some way	45	30
The antidepressant seems to make me/the patient just not care about things that should matter to me/them	40	17
The antidepressant seems to make me/the patient feel emotionally disconnected from people around me/them	42	16
The antidepressant is preventing me/the patient from feeling pleasant emotions	33	13
The antidepressant changes the way that I/the patient experience(s) my/their emotions in a way that is unhelpful (not helpful) to me/them at the moment	34	10
I/the patient have/has considered stopping (or have/has already stopped) my/their antidepressant because of its emotional side effects	39	18

^a^Proportion of respondents selecting ‘agree a little’ or ‘agree’.

HCP, healthcare provider

The mean FAST total score was significantly higher in the patient-reported cohort than in the HCP-assessed cohort in both the acute and remission phases (*p* < 0.01 for both disease phases). Mean (SD) FAST total scores in the acute phase of depression were 47.0 (15.8) points in the patient-reported cohort and 39.1 (15.8) points in the HCP-assessed cohort. Mean (SD) FAST scores in the remission phase were 33.5 (16.1) points versus 19.4 (16.0) points in the two cohorts, respectively. In both cohorts, greatest impairment was seen in the ‘interpersonal relationships’, ‘cognitive functioning’, and ‘occupational functioning’ FAST domains.

### Impact on functioning and quality of life

**Fig. 5:**
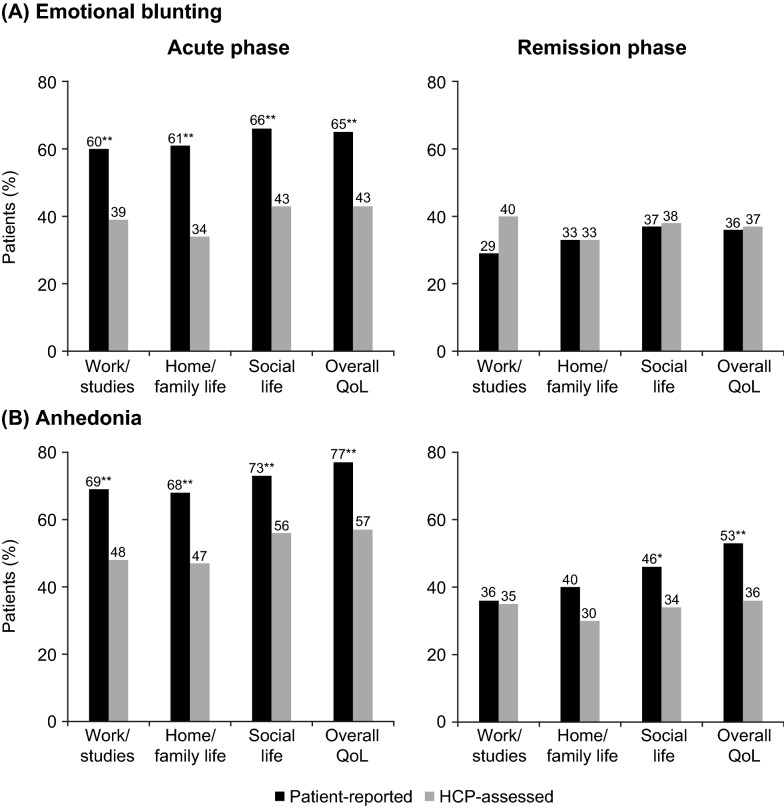
Proportion of patients experiencing a significant impact (i.e., score of 6 or 7 on a scale of 1–7) on functioning and overall quality of life for **A** emotional blunting and **B** anhedonia according to phase of depression. *p < 0.05, **p < 0.01 for patient-reported vs HCP-assessed cohort within phase of depression. Emotional blunting — patient-reported cohort: acute, *n* = 300; remission, *n* = 452. HCP-assessed cohort: acute, *n* = 122; remission: *n* = 52. Anhedonia (i.e., patients experiencing lack of interest or pleasure in activities) — patient-reported cohort: acute, *n* = 231; remission, *n* = 236. HCP-assessed cohort: acute, *n* = 255; remission: *n* = 83.  HCP, healthcare provider; QoL, quality of life

A significantly higher proportion of patients than HCPs reported a significant impact of emotional blunting on all aspects of daily functioning (work, home/family, and social life) and overall quality of life in the acute phase of depression (Fig. 5A). The proportion of patients in the acute phase of depression who experienced a significant impact of emotional blunting on functioning and overall quality of life ranged from 60 to 66% across items in the patient-reported cohort, and from 34 to 43% across items in the HCP-assessed cohort (all differences between the two cohorts, *p* < 0.01). In the remission phase of depression, the proportion of patients experiencing a significant impact of emotional blunting on functioning and overall quality of life was similar in the two cohorts (ranging from 29 to 37% in the patient-reported cohort and from 33 to 40% in the HCP-assessed cohort).

For anhedonia (Fig. 5B), the proportion of patients in the acute phase of depression experiencing a significant impact on functioning and overall quality of life ranged from 68 to 77% in the patient-reported cohort and from 47 to 57% in the HCP-assessed cohort (all differences between the two cohorts, *p* < 0.01). In the remission phase, the proportion of patients experiencing a significant impact of anhedonia on functioning and overall quality of life ranged from 36 to 53% in the patient-reported cohort and from 30 to 36% in the HCP-assessed cohort (difference between cohorts, *p* < 0.05 for social functioning and *p* < 0.01 for overall quality of life).

For all other symptom domains, the proportion of patients reported to be experiencing a significant impact on functioning and overall quality of life was consistently lower in the HCP-assessed cohort than in the patient-reported cohort in both the acute and remission phases of depression (Additional file 1: Table S1). For mood symptoms, respective proportions of patients reported to be experiencing a significant impact on functioning and overall quality of life in the patient-reported and HCP-assessed cohorts ranged from 63 to 75% and from 47 to 60% in the acute phase of depression (all differences between the two cohorts, *p* < 0.01), and from 31 to 50% and from 24 to 30% in the remission phase (*p* < 0.05 for impact on home/family life, social life, and overall quality of life). Respective proportions of patients in the two cohorts reported to be experiencing a significant impact of cognitive symptoms on functioning and overall quality of life were 61–72% versus 35–54% in the acute phase (*p* < 0.01 for impact on home/family life, social life, and overall quality of life), and 33–42% versus 21–29% in the remission phase (*p* < 0.05 for home/family life and *p* < 0.01 for social life and overall quality of life). For fatigue/lack of energy, a significant impact of symptoms on functioning and overall quality of life was reported in 67–75% versus 43–54% of patients in the acute phase of depression (all differences between the two cohorts, *p* < 0.01), and 42–53% versus 23–30% of patients in the remission phase in the patient- and HCP-reported cohorts, respectively (all differences between the two cohorts, *p* < 0.01).

## Discussion

This analysis was undertaken to explore similarities and differences in patient and HCP perspectives concerning the experience and impact of emotional blunting in patients with depression receiving antidepressant therapy. Our findings suggest that HCPs not only substantially underestimate the prevalence of emotional blunting in patients with depression, but also its severity and impact, particularly during the acute phase of the disease. This may at least in part be due to limited use of screening tools for emotional blunting, such as the ODQ screening question or the full ODQ scale, in routine practice settings. In addition, clinical experience suggests that patients likely do not spontaneously report emotional blunting as often as other depressive symptoms, such as insomnia. This could be because patients may not recognize that they are experiencing emotional numbing or blunting as a symptom of depression unless specifically questioned. It could also be because they become indifferent to emotional blunting itself and, therefore, will be unlikely to report it.

By design, all patients completing this survey were experiencing emotional blunting; almost three-quarters of patients in the acute phase of depression and one-quarter of those in remission rated their emotional blunting as extremely severe. According to HCPs, however, only 32% of patients in the acute phase and 14% of those in remission were currently experiencing emotional blunting; this was considered to be extremely severe in 30% of those in the acute phase and 15% of those in remission. Previous studies investigating the prevalence of emotional blunting in depression suggest that approximately 50–60% of patients with MDD receiving treatment with SSRIs or SNRIs experience some degree of emotional blunting [1–7]. As such, our findings clearly indicate that HCPs may underestimate the prevalence of emotional blunting in patients with depression. During the acute phase of the disease, HCPs were more likely than patients to report that emotional blunting was caused by depression. A higher proportion of patients than HCPs considered that their antidepressant medication contributed to their emotional blunting, particularly during the acute phase. This may be because HCPs more traditionally focus on other symptoms of depression and therefore may not consider the need for treatment of blunted positive or negative emotions and/or the effect of antidepressant treatment on positive or negative emotions.

Compared with HCPs, patients perceived a greater impact of emotional blunting on all aspects of functioning and overall health-related quality of life. According to patients, emotional blunting has a greater impact on functioning and quality of life in the acute phase of depression than in the remission phase. In contrast, HCPs reported no difference in the impact of emotional blunting between the acute and remission phases. Irrespective of disease phase, patients also reported a higher prevalence of anhedonia and other core symptoms of depression (i.e., mood, cognitive, and physical [fatigue/lack of energy] symptoms) than HCPs. Patients also reported a greater impact of these symptoms on functioning and quality of life. These differences between patient and HCP perceptions were most apparent in the remission phase, suggesting that HCPs likely underestimate the prevalence of residual symptoms in patients whom they consider to have achieved remission.

Other studies have also shown clinically relevant differences between patient and HCP perceptions in depression [17–21]. This may be of clinical significance, as discordance between patient and physician perceptions has been shown to be associated with poorer treatment outcomes in patients with depression [28]. For example, in another recent online survey, patients with depression reported experiencing a wider range of symptoms, greater impairment of functioning, and different treatment priorities compared with HCPs across all phases of the disease [18, 19]. Indeed, these findings were replicated in the present survey. Mean FAST total scores in the present survey were also broadly similar to those reported in the previous survey for both the patient-reported and HCP-assessed cohorts, with HCPs tending to underestimate the degree of functional impairment experienced by patients in both the acute and remission phases of depression.

An improved understanding of patients’ lived experience of depression is important to ensure that treatment is meaningful and responsive to patients’ needs and to enable them to achieve functional recovery. Our findings suggest that patients presenting with depression should be routinely assessed for emotional blunting using a validated screening question, such as that developed by Price and colleagues [25]: *‘Emotional effects of depression and treatment vary, but may include, for example, feeling emotionally “numbed” or “blunted” in some way; lacking positive emotions or negative emotions; feeling detached from the world around you; or “just not caring” about things that you used to care about. Have you experienced such emotional effects?’ *This simple screening question can be easily used in routine practice settings, permitting rapid identification of patients who are experiencing emotional blunting.

Improved recognition of emotional blunting in patients with major depressive disorder may permit more targeted therapeutic interventions, resulting in improved treatment outcomes. If patients experience blunted emotions with their SSRI or SNRI treatment, alternative antidepressants should be explored to prevent premature discontinuation of necessary treatment and thereby an increased risk of relapse. In one recent study, improvements in emotional blunting observed during treatment with the multimodal antidepressant vortioxetine in patients with depression experiencing residual depressive symptoms and emotional blunting after prior antidepressant therapy were shown to be strongly and significantly correlated with improvements in overall functioning [29]. Mediation analysis showed that almost two-thirds of the improvement in functioning was a direct effect of the improvement in emotional blunting seen after switching to vortioxetine [29].

### Methodologic considerations

To our knowledge, this is the first study to investigate real-world differences between patients and HCPs concerning the experience and impact of emotional blunting across different phases of depression. Potential general study limitations are outlined in detail in the first paper in this series [22]. In terms of this analysis, comparisons between the patient and HCP cohorts should be interpreted with caution as responses were not paired. It should also be taken into consideration that both patients and clinicians responded to questions based on the FAST, which was developed as a clinician-reported assessment tool. Similarly, the potential relationship of emotional blunting with the underlying depression and antidepressant treatment was assessed in both cohorts using questions from the ODQ, which was developed for patient use.

## Conclusion

The results of this survey suggest that HCPs may underestimate the prevalence and severity of emotional blunting in patients with depression, as well as its impact on patients’ overall functioning and health-related quality of life, particularly in the acute phase of the disease. The risk of premature treatment discontinuation as a result of emotional blunting also appears to be underappreciated by HCPs. These findings highlight the importance of recognizing and treating emotional blunting in patients with depression to help them achieve not only remission from their symptoms, but also full functional recovery.

## Supplementary Information

**Additional file 1: Table S1** Percentage of patients experiencing a significant impact (score of 6 or 7 on a scale of 1–7) of mood symptoms, cognitive symptoms, and fatigue/lack of energy on functioning and overall quality of life by phase of depression.

## Data Availability

The datasets presented in this article are not readily available given the informed consent provided by survey participants. Requests to access the datasets should be directed to the authors.
