# Supplementary material for: Emotional blunting in patients with depression. Part IV: differences between patient and physician perceptions
Source: Ann Gen Psychiatry. 2022 Jun 22;21:22. doi: 10.1186/s12991-022-00391-5 (PMC9215037; doi:10.1186/s12991-022-00391-5)
Supplement: Supplementary file 1 — Additional file 1: Table S1 Percentage of patients experiencing a significant impact (score of 6 or 7 on a scale of 1–7) of mood symptoms, cognitive symptoms, and fatigue/lack of energy on functioning and overall quality of life by phase of depression. [file 12991_2022_391_MOESM1_ESM.pdf]

Additional file 1

Table S1 Percentage of patients experiencing a significant impact (score of 6 or 7 on a scale of 1–7) of mood symptoms, cognitive symptoms, and fatigue/lack of energy on functioning and overall quality of life by phase of depression.

Symptom domain	Acute		Remission	
	**Patient-reported cohort**	**HCP-assessed cohort**	**Patient-reported cohort**	**HCP-assessed cohort**
**Mood symptoms**	(*n* = 250)	(*n* = 296)	(*n* = 280)	(*n* = 102)
Impact on work/studies	63	52**	31	28
Impact on home/family life	65	47**	37	24*
Impact on social life	69	54**	41	28*
Impact on overall quality of life	75	60**	50	30*
**Cognitive symptoms**	(*n* = 220)	(*n* = 237)	(*n* = 252)	(*n* = 121)
Impact on work/studies	61	54	38	29
Impact on home/family life	62	35**	33	21*
Impact on social life	63	40**	36	22**
Impact on overall quality of life	72	47**	42	21**
**Fatigue/lack of energy**	(*n* = 253)	(*n* = 298)	(*n* = 317)	(*n* = 169)
Impact on work/studies	67	51**	44	30**
Impact on home/family life	68	43**	42	26**
Impact on social life	72	45**	48	23**
Impact on overall quality of life	75	54**	53	30**

*p < 0.05; **p < 0.01 for patient-reported vs HCP-assessed cohort within phase of depression.
HCP, healthcare provider; *n,* number of patients reporting symptoms.
